# c-Rel is a Novel Oncogene in Lung Squamous Cell Carcinoma Regulating Cell Proliferation and Migration

**DOI:** 10.7150/jca.93766

**Published:** 2024-03-04

**Authors:** Renru Luo, Qiongyu Liu, Zheyu Hu, Wanqin Dai, Shuwei Huang, Jianjiang Xie, Shuquan Wei, Chuwen Lin

**Affiliations:** 1 Department of histology and embryology, School of Medicine, Shenzhen Campus of Sun Yat-Sen University, Sun Yat-Sen University, Shenzhen, Guangdong, China.; 2 Department of Pulmonary and Critical Care Medicine, Guangzhou First People's Hospital, School of Medicine, South China University of Technology, Guangzhou, Guangdong, China.; 3 School of Ophthalmology and Optometry, Wenzhou Medical University, Wenzhou, Zhejiang, China.

**Keywords:** c-Rel, REL, NFκB pathway, LUSC.

## Abstract

Lung squamous cell carcinoma (LUSC) accounts for approximately 25% to 30% of lung cancers, but largely no targeted therapy is available against it, calling for identification of new oncogenes in LUSC growth for new therapeutic targets. In this study, REL was identified through a screening for oncogenes that are highly amplified in human LUSC. Its expression was associated with poor prognosis in LUSC patients. Furthermore, knockdown of c-Rel in LUSC cell lines lead to significant decrease in cell proliferation and migration. Mechanistically, c-Rel knockdown suppressed NFκB pathway by blocking phosphorylation of IκB. Consistently, pharmaceutic inhibition of c-Rel also. In orthotopic xenograft lung cancer mouse model, c-Rel knockdown inhibited the tumor growth. Cancer cell proliferation and epithelial-mesenchymal-transition (EMT) of the tumors were impaired by c-Rel knockdown. Finally, it's confirmed in precision-cut tumor slices of LUSC that deletion of c-Rel inhibits the NFκB pathway and cancer cell growth. Accordingly, we hypothesize that c-Rel promotes the activation of the NFκB pathway by promoting the phosphorylation of IκB in LUSC. Our study reveals REL as a novel LUSC oncogene and provides new insights into the molecular regulation of LUSC, which will provide new therapeutic targets for the treatment of squamous lung cancer.

## 1. Introduction

Lung squamous cell carcinoma (LUSC) is a subtype of non-small cell lung cancer (NSCLC) that accounts for approximately 30% of all lung cancers and contributes to over 400,000 deaths globally per year^1^. Although targeted therapies such as epidermal growth factor receptor (EGFR) inhibitors have exhibited efficacy against lung adenocarcinoma, there are no first-line targeted therapies available for LUSC^2^. Consequently, the current 5-year survival rate for LUSC is lower than 20%^3^. Thus, there is an urgent need to identify new oncogenes that promotes LUSC growth, which are ideal candidates for therapeutic targets against LUSC.

The nuclear factor-κB (NFκB) family comprises a group of dimeric transcription factors including RelA/p65, RelB, c-Rel, p50/p105, and p52/p100, which exhibit both shared and divergent biological functions. NFκB complex usually exist in an inactive state bound to inhibitory proteins called inhibitors of NFκB (IκBs)^4^. Upon activation, IκB undergoes phosphorylation and rapid degradation, allowing the translocation of NFκB to the nucleus, where it elicits genes that controls multiple processes involved in cancer pathogenesis including proliferation, apoptosis, angiogenesis, and metastasis^5^. Beside a well-established role in regulating inflammation, a large body of evidence has demonstrated that NFκB pathway plays an essential role in cancer development and progression. However, the role of NFκB pathway in cancer is complex and multifaceted. NFκB pathway function both in tumor cells and in the tumor microenvironment^6^. The multilayered roles and opposing functions of NFκB pathway in cancer have hindered the clinical translation of NFκB-targeted therapies^6^.

c-Rel is encoded by the proto-oncogene REL gene in human^7^. It has important roles in lymphoid cell functions, and in hematopoietic malignancies. Amplifications of REL in genome, which are often associated with elevated levels of nuclear c-Rel protein, have been detected in large portion of lymphoma patients^8^. c-Rel is also implicated in breast, head and neck, and pancreatic cancers, but overall its functions in solid tumors is not well explored, and in particular its function in LUSC is unknown^9^-^12^. Mechanistically, it has been demonstrated that REL promotes tumor growth by inducing the generation of regulatory T cells (Tregs) and the differentiation of myeloid-derived suppressor cells (MDSCs) in immune cells^13^, ^14^. Nevertheless, the effect of c-Rel on tumor cells themselves has yet to be fully elucidated. If c-Rel in tumor cells also promotes tumor growth as they do in immune cells, it may serve as a promising target for therapeutic interventions against cancer.

Here we conducted a screening for oncogenes of LUSC and REL was identified. The expression of REL was associated with poor survival of LUSC patients. Importantly, silencing of c-Rel reduces LUSC cell growth by inhibiting cell proliferation and migration. Mechanistical investigation revealed inactivation of NFκB pathway in c-Rel-silenced LUSC cell lines. Pharmaceutic inhibition of c-Rel suppressed LUSC cell growth. Orthotopic xenograft mouse model and human precision-cut lung slices model further confirmed c-Rel promoted LUSC growth. Our study identified a new oncogene of LUSC, shed new light on the mechanisms regulating LUSC growth, and pave the way for developing new pharmaceutic targets against the disease.

## 2. Materials and Methods

### 2.1. Patient tumor collection

The specimens of surgically excised tumors were procured from the Guangzhou First People's Hospital situated in China. The patients included in this study had not undergone any form of treatment prior to surgery, thus ensuring the purity of the tissue samples and the integrity of our findings. The study protocol adhered strictly to the ethical guidelines set forth by the esteemed Ethics Committee of the School of Medicine at Sun Yat-Sen University. Moreover, prior to participating in the study, each patient provided informed consent.

### 2.2. Animal

The C57BL/6 mice used in this study were bred and housed in a specific pathogen-free animal facility at Sun Yat-Sen University. The mice were kept in a controlled 12-hour light and 12-hour dark cycle. Random assignment of the mice to different experimental groups was done for follow-up experiments. All animal procedures conducted in this study were performed in compliance with the guidelines for the treatment of laboratory animals and approved by the Institutional Animal Care and Use Committee of Sun Yat-Sen University.

### 2.3. Lewis lung cancer model

A DMEM medium (C11995500BT, Gibco) containing 10% fetal bovine serum (FBS) (10271106, Gibco) and 1% penicillin-streptomycin (PS) (BL505A, Biosharp) was used for maintaining Lewis cells. And a 37°C incubator with 5% CO_2_ was used to maintain all cells.

Lewis cells (2×10^6^ cells) suspended in 100 μl phosphate-buffered saline (PBS) were injected into the lung of the C57BL/6 mice by intratracheal administration (i.t.), then harvested at 21 days post injection (dpi).

### 2.4. Cell culture

SK-MES-1 cells were maintained in MEM medium (C11095500BT, Gibco) augmented with 1% PS (BL505A, Biosharp) and 10% FBS (10270106, Gibco).

NCI-H1703 cells were maintained in RPMI-1640 medium (C11875500BT, Gibco) augmented with 1% PS (BL505A, Biosharp) and 10% FBS (10270106, Gibco). To emulate the physiological environment, the cells were incubated in a CO_2_-rich environment (5% CO_2_) at a constant temperature of 37°C.

### 2.5. Human precision-cut lung slices (PCLS) culture

Human tissues were carefully embedded in 2% low melting agarose (16520050, Thermo). Subsequently, the agarose-embedded tissues were mounted on a specimen holder in preparation for the slicing process. The precision-cut vibrating-blade microtome, specifically the Leica VT1200S model, was employed to accurately cut the tissue slices at a thickness of 200 μm. Following the slicing procedure, the PCLS were incubated in 24-well plates with DMEM/F-12 medium (C11330500BT, Gibco) supplemented with 1% PS (BL505A, Biosharp). The provision of optimal environmental conditions necessitated the placement of the PCLS within an incubator, maintained at a temperature of 37°C and a 5% CO_2_ atmosphere.

To enable the conduction of detailed histological analysis, the sections were fixed in 1% paraformaldehyde (PFA) for 15 minutes and paraffin sectioned 48 hours after lentivirus infection.

### 2.6. Lentivirus-mediated shRNA and lentiviral infection

Lentivirus-mediated shRNA was generated by cloning oligonucleotides encoding shRNA into pLentiLox3.7 vector (Addgene#11795).

For virus production, vectors containing targeting sequences were transfected in HEK293T cells together with packaging vectors pMD2.G (Addgene #12259) and psPAX2 (Addgene#12260) using polyethylenimine (PEI) (23966-1, Polysciences). Viruses were harvested at 48 hours post-transfection and filtered through a 0.45 μm filter.

For infection, 250 μl lentivirus was added to the cells with 2 μg polybrene (sc-134220, Santa) per well. Media were changed 6 hours after infection.

### 2.7. RNA extraction and quantitative RT-PCR

Total RNA isolation from lung tissues or cells was accomplished employing the TRIzol reagent (15596018, Invitrogen), following the stipulated protocols of the manufacturer. The synthesis of complementary DNA (cDNA) was conducted using the HiScript III All-in-one RT SuperMix Perfect for qPCR Kit (R333, Vazyme). Quantification of gene expression was conducted using real-time PCR with gene-specific primers and SYBR Green probes (Q712, Vazyme), and assessment was performed utilizing an HT7900 quantitative PCR machine (Applied Biosystems). The list of primers used can be found in Table S1 of the Supplementary Information Appendix. The normalization of gene expression was achieved by employing the expression levels of the house-keeping gene Actin as reference.

### 2.8. Cell counting kit-8 assay

Cells were then plated into individual compartments of a 96-well plate at a density of 1×10^4^ cells per well. These cultured cells were then subjected to time-dependent conditions of 0h, 24h, 48h and 72h, with triplicate replicates for each group. The CCK8 kit (#K1018, APExBio) was used to assess the level of cellular viability according to the careful guidelines provided by the manufacturer.

### 2.9. Wound healing assay

Cells were inoculated into 24-well plate 48 hours after transfection. When the cell confluence reached 90%, the cells were scratched in the shape of "+" using a 200 μl pipette tip. After washing the cells three times with PBS, serum-free medium was added for migration for 0h, 24h, 48h and 72h.

### 2.10. Lung histopathological analysis

Lung tissues were fixed in 1% PFA for 1 hour, and then embedded in paraffin for sectioning. H&E staining were performed for histopathological analysis.

### 2.11. 5-Ethynyl-2'-deoxyuridine (EdU) staining

Cell climbing was placed in the 24-well plate, and 2 × 10^5^ cells were inoculated into the 24-well plate and cultured overnight. 2 hours before harvest, we added EdU solution (10 μM/ml) and incubated for 20 minutes. In accordance with the manufacturer's directions, EdU Imaging Kits (K1075, Apexbio) was used to stain the proliferative cells.

### 2.12. Western blot analysis

Lysing of lung tissues and cells was carried out in cold RIPA Lysis Buffer (Beyotime) supplemented with phosphatase inhibitor cocktail (Beyotime) and protease inhibitor cocktail (Beyotime). After incubation for 30 minutes with rotating in 4°C, supernatants were collected after centrifugation (15,000 rpm, 15 minutes, 4°C) and protein concentrations was determined by using BCA Protein Assay (Beyotime). Total protein was denatured by SDS-PAGE and then transferred from the gel to the activated NC membranes. After blocked with 5% milk, the NC membrane was hybridized overnight with appropriate primary antibodies (P65, #32705, Signalway Antibody; IκBα, #4814, Cell Signaling Technology; p-P65, MAB72261, R&D Systems; p-IκBα, # 2859, Cell Signaling Technology; E-cadherin, 610181, BD Biosciences; vimentin, #5741, Cell Signaling Technology; β-actin, #4970, Cell Signaling Technology). A fluorescent secondary antibody (LI-COR) was incubated on the membranes, and then Odyssey imaging system (LI-COR) was used to scan the membranes after they had been washed.

### 2.13. Immunofluorescence staining

Cells were fixed on coverslips for 10 minutes at room temperature (RT) using 1% PFA solution. PBS was used as wash medium, followed by 0.2% Triton X-100 in PBS as permeabilizer for 20 minutes and 5% donkey serum/0.1% Triton X-100/PBS as blocking solution. In order to specifically target the protein of interest, the cells were then subjected to an incubation at 4°C with rabbit anti-P65 antibody (#32705, Signalway Antibody) overnight. Cells were gently washed with PBS on the following day.

And then the cells were incubated with donkey anti-rabbit Alexa Fluor 488 (ab150073, Abcam) and DAPI (#C0060, Solarbio). Finally, the prepared sections were mounted and subsequently imaged using a fluorescence microscope (Zeiss Axio Observer 7).

### 2.14. Data mining

The data of lung cancer gene comparison was from TCGA data curated by UALCAN database (http://ualcan.path.uab.edu/cgi-bin/ualcan-res.pl), cBioPortal for Cancer Genomics (https://www.cbioportal.org/mutation_mapper) and human protein atlas (https://www.proteinatlas.org/).

### 2.15. Statistical analysis

Data was presented in this study as the mean ± standard deviation (SD). To determine the statistical significance of the results, GraphPad Prism 9.2.0 was used for statistical comparisons. And data were subjected using either two-way ANOVA or paired two-tailed Student's t test. The level of statistical significance is indicated by the mean ± SD as follows: p < 0.05 (*), p < 0.01 (**), p < 0.001 (***), and p < 0.0001 (****).

## 3. Results

### 3.1. c-Rel was identified in a screening for oncogenes amplified in LUSC

**Figure 1:**
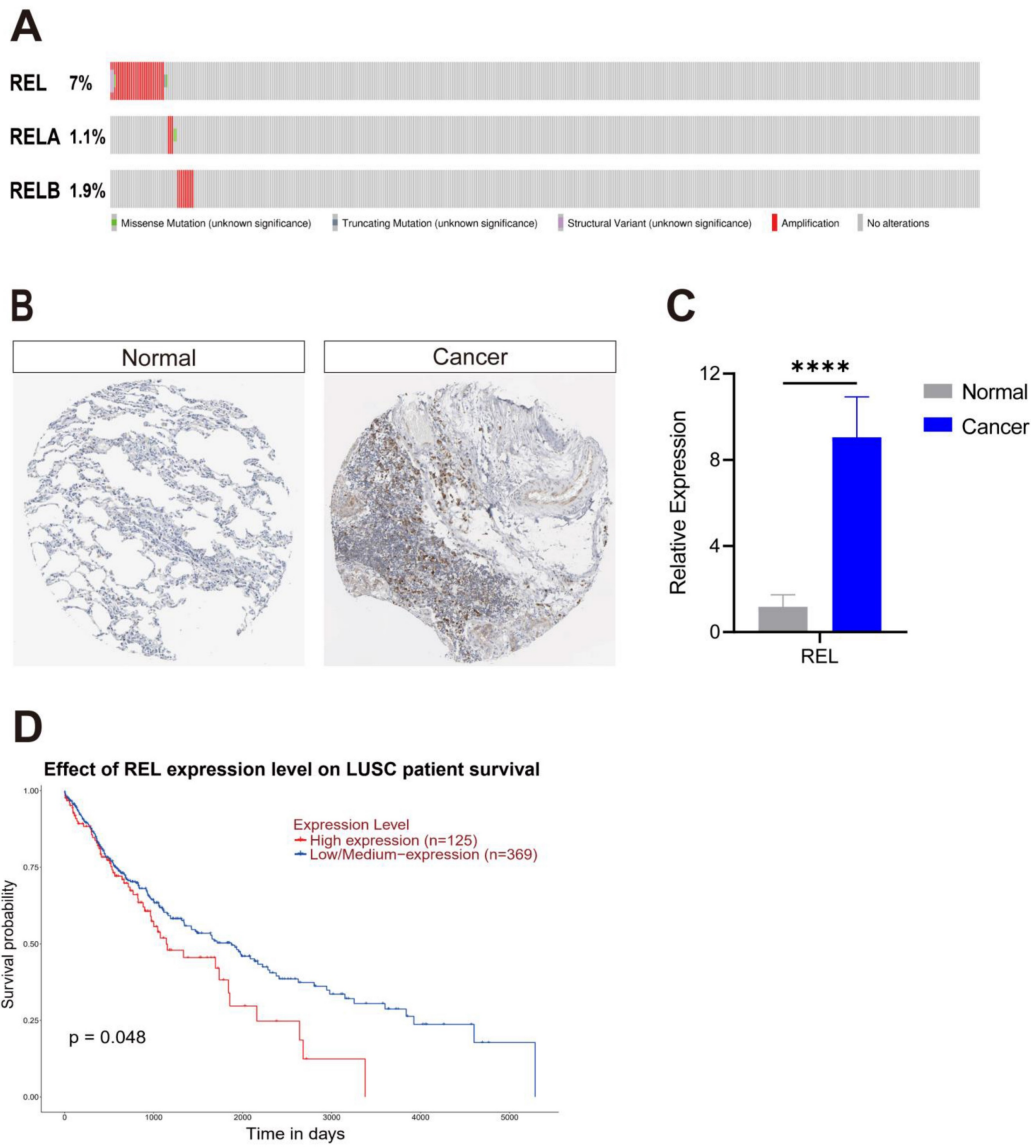
** c-Rel was identified in a screening for oncogenes amplified in LUSC. (A)** The TCGA dataset highlights the amplification of REL, RELA, and RELB in LUSC. **(B)** Expression of c-Rel protein in Health (Normal) and LUSC (Cancer) from Human Protein Atlas database. **(C)** A qPCR assay was performed to measure REL expression in non-cancerous lung tissues (Normal) and LUSC tissues (Cancer) obtained from LUSC patients (N=5). ****, p<0.0001. **(D)** Comparative analysis of overall survival data derived from the TCGA database demonstrated a difference in survival rates between patients with low and high REL expression levels.

A characteristic of lung squamous cell carcinoma (LUSC) in the genomic landscape compared to lung adenocarcinoma is that LUSC possess far more copy number variations (CNV) including both amplification and deletion, and much less mutations. Thus, in order to identify new pharmaceutical targets in LUSC, we performed an integrative screening for oncogenes that are highly amplified in LUSC. Genes that exhibit high level amplification of copy numbers in >5% LUSC patients were selected using datasets curated in cBioPortal. Among these hits, genes whose expression is associated with poor prognostic were selected using data provided by PRECOG. Among the selected ones, 25 Tier 1 oncogenes were selected based on the Cancer Gene Census in COSMIC database. Among the 25 genes, 7 are oncogenes that were previously implicated in LUSC including EGFR, PIK3CA, and SOX2 etc. The rest 18 genes were never directly implicated in LUSC, among which is REL.

c-Rel was amplified in 7% of LUSC cases (Figure 1A). Immunohistochemistry data curated by Human Protein Atlas database showed its encoded protein c-Rel was significantly increased in human LUSC samples (Figure 1B). We examined its mRNA expression in LUSC samples from patients by quantitative PCR, and consistently, REL was upregulated in LUSC compared to that in the paracancerous tissues (Figure 1C). Survival analysis of lung cancer data in TCGA revealed that LUSC patients with high REL expression exhibited a significantly lower survival rate (Figure 1D). Altogether these data suggested high REL is an oncogene of LUSC.

### 3.2. c-Rel promoted LUSC growth by enhancing cell proliferation and migration

REL encoded c-Rel, a transcription factor of the canonical NFκB pathway. It's a prototype of oncogene that play important roles in hematopoietic and some solid malignancies. Its role in LUSC, however, is never shown. In contrast to REL, REL A and RELB which encoded another two NFκB transcription factors, was only amplified in <2% LUSC (Figure 1A). Moreover, none of their expression was significantly associated with LUSC patient survival (Figure S1). This suggests that c-Rel is the major NFκB effector that modulate LUSC growth.

**Figure 2:**
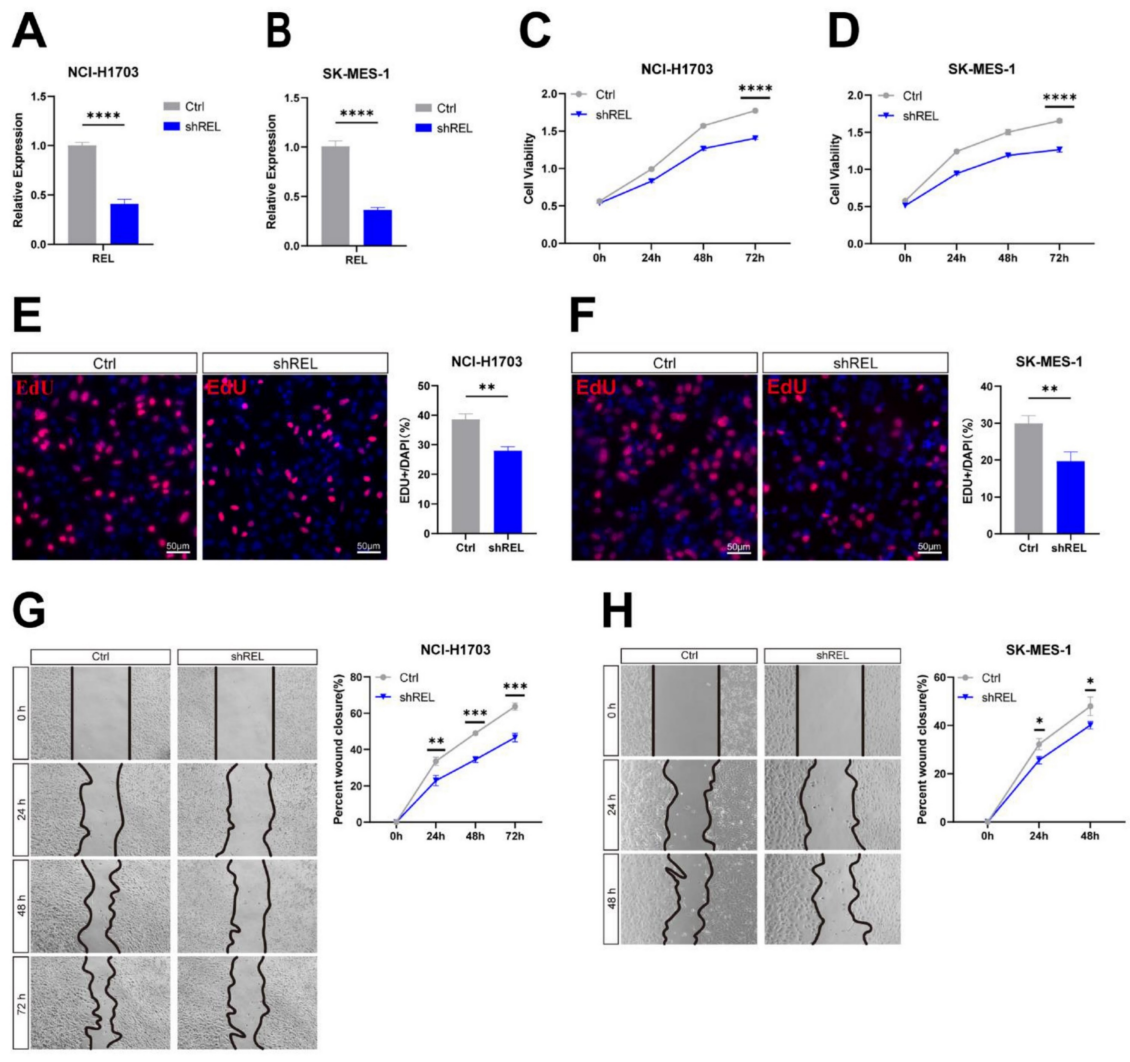
** c-Rel promoted LUSC growth by enhancing cell proliferation and migration. (A)** The expression level of REL was analyzed using qPCR in NCI-H1703 cells without (Ctrl) and with c-Rel knockdown (shREL) (N=3). ****, p<0.0001. **(B)** The expression level of REL was analyzed using qPCR in SK-MES-1 cells without (Ctrl) and with c-Rel knockdown (shREL) (N=3). ****, p<0.0001. **(C)** Cell viability in NCI-H1703 cells without and with c-Rel knockdown was assessed using the CCK8 assay (N=3). ****, p<0.0001. **(D)** Cell viability in SK-MES-1 cells without and with c-Rel knockdown was assessed using the CCK8 assay (N=3). ****, p<0.0001. **(E)** Immunofluorescence staining (IF) for the proliferation marker EdU (red) was performed to evaluate cell proliferation in NCI-H1703 cells without and with c-Rel knockdown (N=5). **, p<0.01. Scale bars, 50 μm. **(F)** IF for the proliferation marker EdU (red) was performed to evaluate cell proliferation in SK-MES-1 cells without and with c-Rel knockdown (N=5). **, p<0.01. Scale bars, 50 μm. **(G)** The migratory potential of NCI-H1703 cells without and with c-Rel knockdown was assessed using scratch assay (N=3). **, p<0.01; ***, p<0.001. **(H)** The migratory potential of SK-MES-1 cells without and with c-Rel knockdown was assessed using scratch assay (N=3). *, p<0.05.

To directly interrogate c-Rel function in LUSC, we silenced c-Rel by shRNA (shREL) in LUSC cell line NCI-H1703 and SK-MES-1(Figure 2A and 2B). Cell viability assay showed that silencing of c-Rel significantly inhibits cancer cell growth (Figure 2C and 2D). EdU assay revealed decreased proliferation upon c-Rel knockdown (Figure 2E and 2F). Moreover, the migration ability was also reduced upon c-Rel knockdown as scratch assay demonstrated (Figure 2G and 2H). Thus, c-Rel strengthens LUSC growth by promoting cell proliferation and migration.

### 3.3. c-Rel regulated LUSC growth through activating NFκB pathway

**Figure 3:**
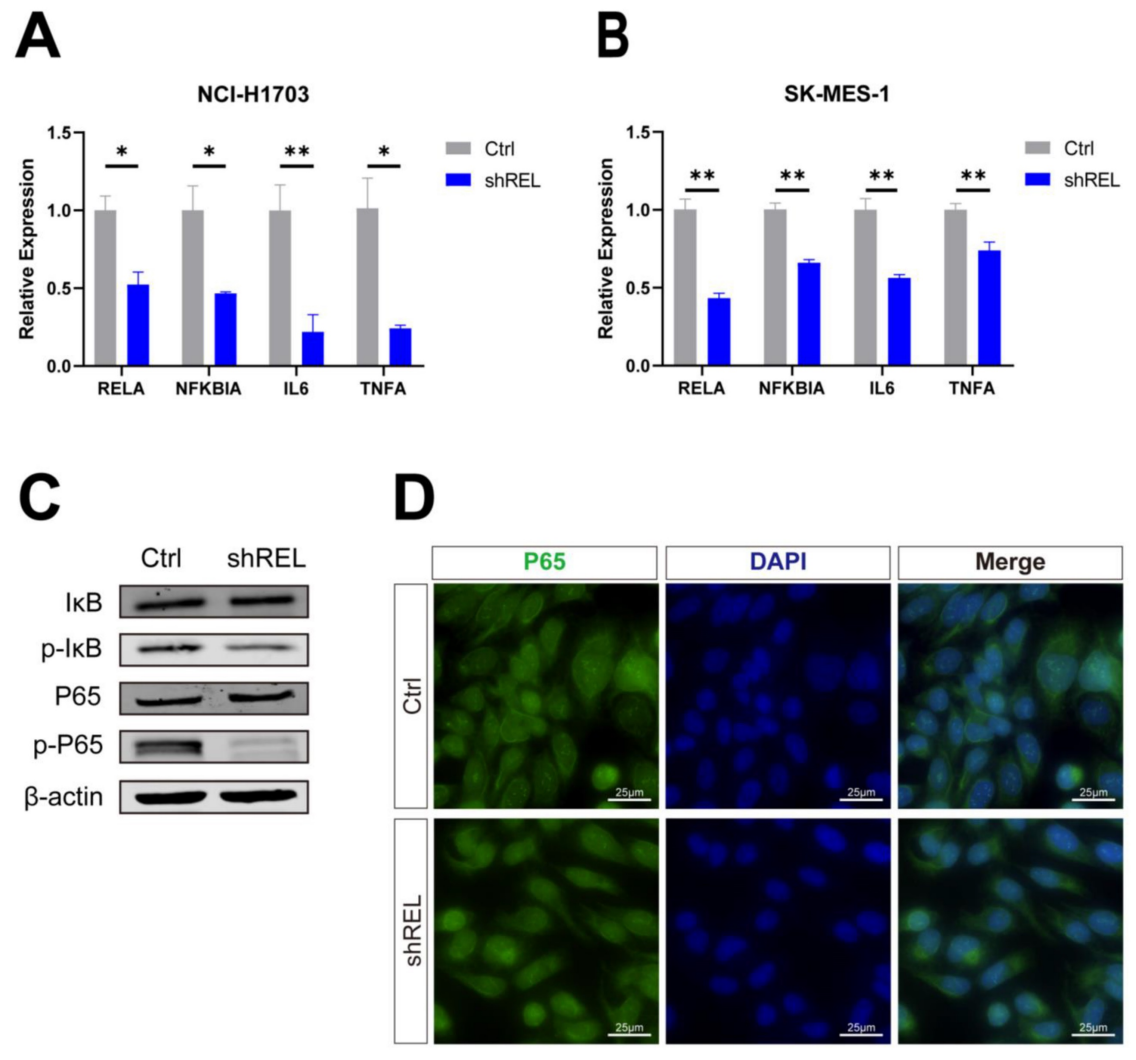
** c-Rel regulated LUSC growth through activating NFκB pathway. (A)** The expression level of NFκB pathway target genes in NCI-H1703 cells without (Ctrl) and with c-Rel knockdown (shREL) using qPCR (N=3). *, p<0.05; **, p<0.01. **(B)** The expression level of NFκB pathway target genes in SK-MES-1 cells without and with c-Rel knockdown using qPCR (N=3). **, p<0.01. **(C)** The protein level of IκB, p-IκB, P65, and p-P65 were examined by western blot in NCI-H1703 cells lysates without and with c-Rel knockdown. **(D)** IF for P65 (green) in NCI-H1703 cells lysates without and with c-Rel knockdown. Scale bars, 25 μm.

We next investigated the molecular mechanisms via which c-Rel regulates LUSC growth. As a key component of NFκB pathway, we speculated that c-Rel would modulate tumor growth through this pathway. Indeed, target genes of NFκB pathway were downregulated upon c-Rel silencing in NCI-H1703 and SK-MES-1 (Figure 3A and 3B). Phosphorylation of IκB leads to its degradation and thus promoted P65 phosphorylation and activation. In c-Rel knockdown cells, the level of phosphorylated IκB was decreased, indicating blocked phosphorylation of IκB (Figure 3C). Consistently, phosphorylation of P65 was also decreased (Figure 3C). In accordance, immunostaining showed that P65 accumulated in the nucleus of WT cells, but the nuclear P65 was reduced in cells with c-Rel silencing (Figure 3D). Altogether these results demonstrated that the activity of NFκB pathway was suppressed upon c-Rel knockdown.

### 3.4. Pharmaceutical inhibition of c-Rel attenuated LUSC growth

**Figure 4:**
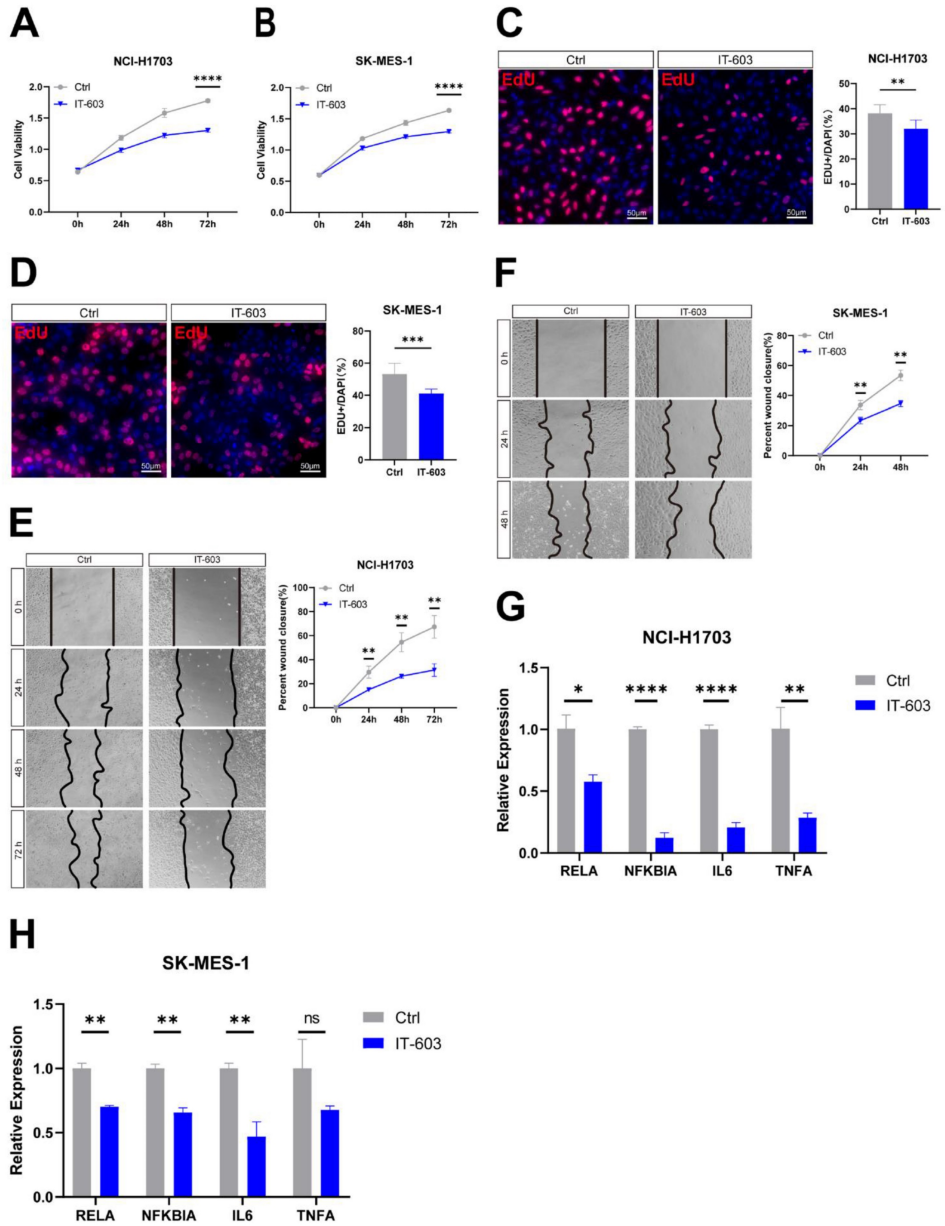
** Pharmaceutical inhibition of c-Rel attenuated LUSC growth. (A)** Cell viability in DMSO (Ctrl) and IT-603-treated (IT-603) NCI-H1703 cells was assessed using the CCK8 assay (N=3). ****, p<0.0001. **(B)** Cell viability in DMSO (Ctrl) and IT-603-treated (IT-603) SK-MES-1 cells was assessed using the CCK8 assay (N=3). ****, p<0.0001. **(C)** IF for the proliferation marker EdU (red) was performed to evaluate cell proliferation in DMSO and IT-603-treated NCI-H1703 cells (N=5). **, p<0.01. Scale bars, 50 μm. **(D)** IF for the proliferation marker EdU (red) was performed to evaluate cell proliferation in DMSO and IT-603-treated SK-MES-1 cells (N=5). ***, p<0.001. Scale bars, 50 μm. **(E)** The migratory potential of DMSO and IT-603-treated NCI-H1703 cells was assessed using scratch assay (N=3). **, p<0.01. **(F)** The migratory potential of DMSO and IT-603-treated SK-MES-1 cells was assessed using scratch assay (N=3). **, p<0.01. **(G)** The expression level of NFκB pathway target genes in DMSO and IT-603-treated NCI-H1703 cells using qPCR (N=3). *, p<0.05; **, p<0.01; ****, p<0.0001. **(H)** The expression level of NFκB pathway target genes in DMSO and IT-603-treated SK-MES-1 cells using qPCR (N=3). ns, not significant; **, p<0.01.

We next examined whether inhibition of c-Rel with small molecule compound could inhibit LUSC growth. IT-603 is a specific c-Rel inhibitor that is a promising modulator of T-cell responses in treating graft-versus-host disease (GVHD)^5^. Treatment of NCI-H1703 and SK-MES-1 cells with IT-603 resulted in a significant decrease in cell viability (Figure 4A and 4B). IT-603 treatment in both cell lines markedly reduced cell proliferation (Figure 4C and 4D), as well as cell migration (Figure 4E and 4F). Consistently, target genes of NFκB pathway activity were downregulated (Figure 4G and 4H), indicating suppressed NFκB pathway. These results indicated that pharmaceutical inhibition of c-Rel could impede LUSC growth through blocking NFκB pathway.

### 3.5. c-Rel promoted LUSC growth *in vivo*

**Figure 5:**
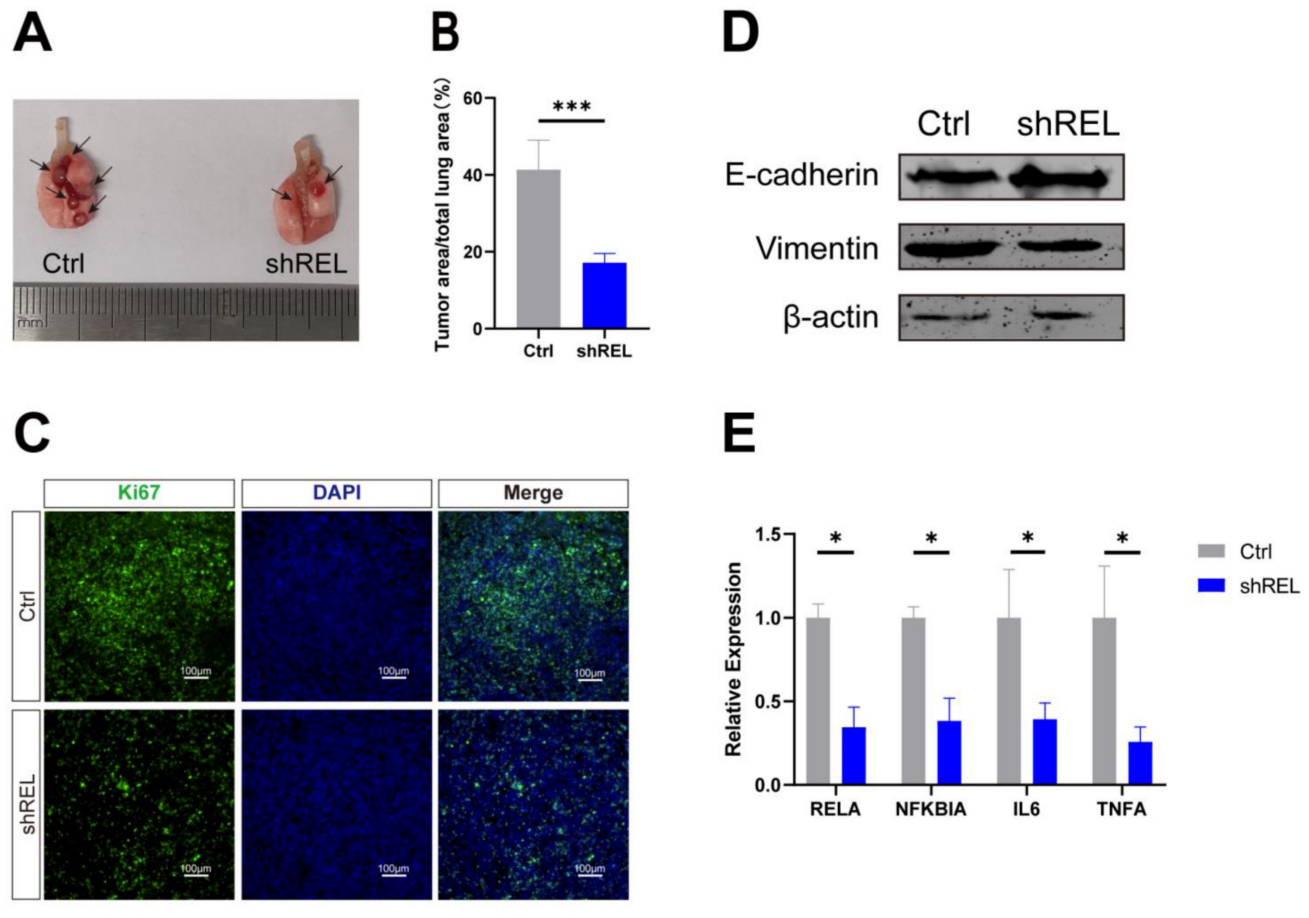
** c-Rel promoted LUSC growth *in vivo*. (A, B)** The number **(A)** and area **(B)** of tumors in the lungs of mice after injection of control (Ctrl) and c-Rel-knockdown (shREL) LLC cells. ***, p<0.001. **(C)** IF for proliferation marker Ki67 (green) in the lungs of mice after injection of control and shREL LLC cells. Scale bars, 100 μm. **(D)** The protein level of E-cadherin and vimentin were examined by western blot in the lungs of mice after injection of control and shREL LLC cell lysates. **(E)** The qPCR analysis of expression level of NFκB pathway target genes in the lungs of mice after injection of control and shREL LLC cells. (N=3). *, p<0.05.

To determine the effect of c-Rel on LUSC *in vivo*, orthotopic xenograft tumor model was generated by intratracheal injection of Lewis lung carcinoma (LLC) cells with silenced c-Rel into the lungs of wild-type C57BL/6 mice. Upon c-Rel silencing, the number and size of tumors in the mice were markedly reduced compared to those in control (Figure 5A and 5B), indicating lower tumor burden upon c-Rel silencing *in vivo*. IF revealed reduced proliferation marker Ki67 in the lungs with c-Rel knockdown, indicating that c-Rel knockdown inhibited the proliferation of tumor cells (Figure 5C). Tumors with c-Rel knockdown expressed more E-cadherin and less vimentin than control, indicating reduced epithelial-mesenchymal-transition (EMT) (Figure 5D). Mechanistically, the target genes of NFκB pathway were downregulated upon c-Rel knockdown (Figure 5E), indicating decreased NFκB pathway activity.

### 3.6. c-Rel promoted LUSC growth in human PCLS

**Figure 6:**
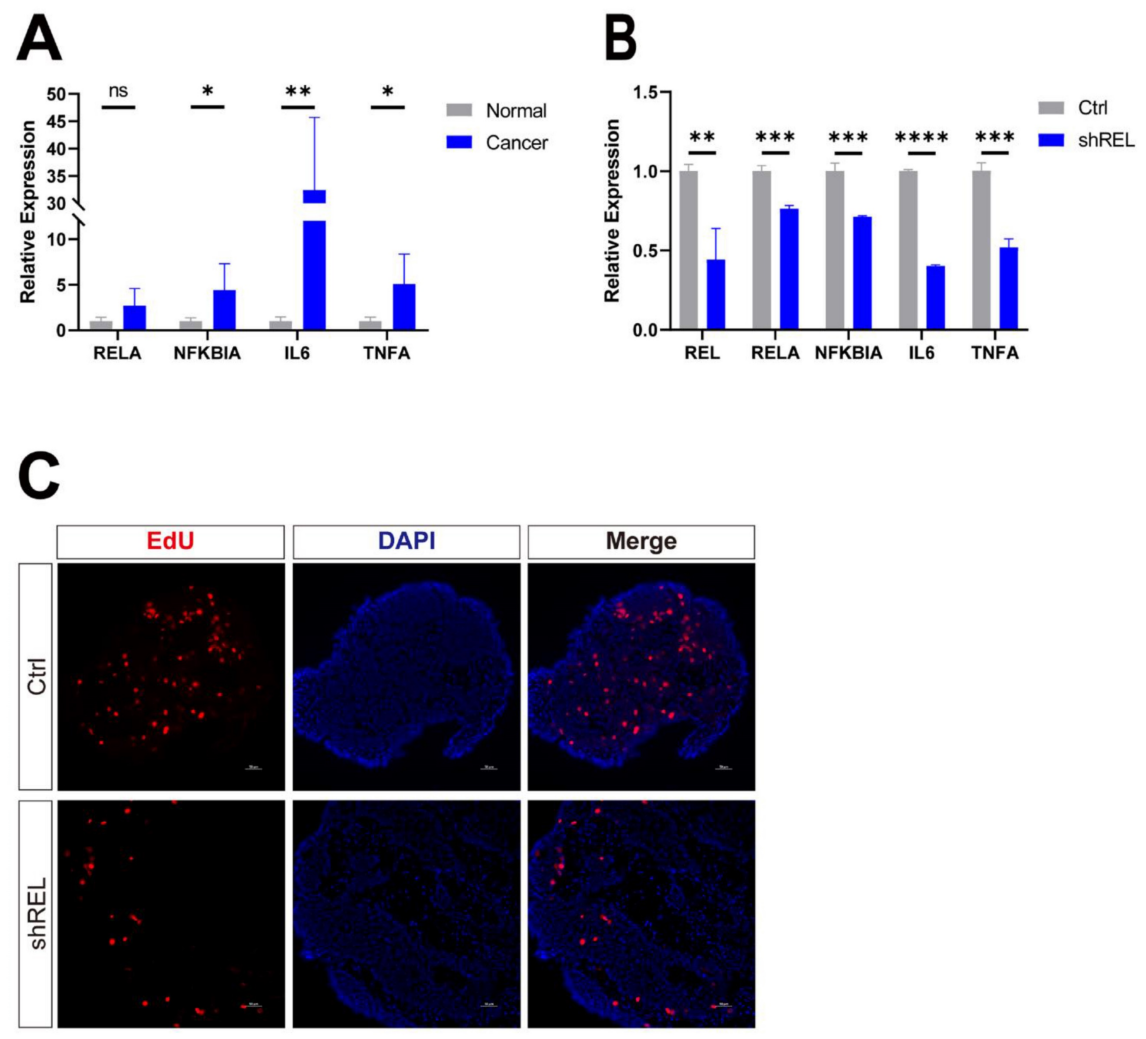
** c-Rel promoted LUSC growth in human PCLS. (A)** The qPCR analysis of expression level of NFκB pathway target genes in non-cancerous lung tissues and LUSC tissues from LUSC patient (N=5). ns, not significant; *, p<0.05; **, p<0.01. **(B)** The qPCR analysis of expression level of NFκB pathway target genes in control and shREL human tumor PCLS. **, p<0.01; ***, p<0.001; ****, p<0.0001. **(C)** IF for proliferation marker EdU (red) in PCLS without (Ctrl) and with c-Rel knockdown (shREL). Scale bars, 50 μm.

Next, we seek to confirm the effect of c-Rel on LUSC in precision-cut lung slices (PCLS). It's a 3D ex vivo model prepared from human LUSC samples which at least partially reflects the complexity of the endogenous environment tumor cells reside in. The target genes of NFκB pathway were upregulated in LUSC compared to that in the paracancerous tissues (Figure 6A). Dysregulation of NFκB pathway target genes was suppressed by silencing of c-Rel in PCLS (Figure 6B). Silencing of c-Rel in PCLS also significantly reduced tumor cell proliferation labelled by EdU (Figure 6C). Therefore, c-Rel promoted tumor growth by activating the NFκB pathway in PCLS of human LUSC.

## 4. Discussion

This study uncovers a new LUSC oncogene REL and demonstrates its function in LUSC. REL was identified through a screen for oncogenes with CNV in LUSC. Its overexpression was associated with poor survival of LUSC patients. Silencing of c-Rel and pharmaceutic inhibition of c-Rel *in vitro* both hindered LUSC cell growth by blocking cell proliferation and migration. Furthermore, this effect was confirmed in orthotopic xenograft mouse model and human PCLS model. These results indicate c-Rel is a promising candidate of pharmaceutic target against LUSC.

This established c-Rel function in tumor cell per se. c-Rel plays roles in multiple processes in tumorigenesis, particularly in immunity regulation and tumor microenvironment^15^, ^16^. c-Rel is known to important roles in blood cancers and its overexpression is also implicated in several solid tumors^17^, ^18^. But its reported functions in cancers was mainly targeting tumor immunity. It is essential for Treg differentiation; moreover, it's required for MDSCs formation, both of which are major components of an immunity-suppressive tumor microenvironment^13^, ^14^. However, its role in tumor cells per se remained a matter of debate. The current study demonstrates c-Rel in tumor cells promotes tumor growth by activating cell proliferation and migration, by assessing its function in cell lines, orthotopic xenograft mouse model, and human PCLS.

Despite its important functions in tumor development and growth, the complex role of NFκB pathway in cancer has hindered the clinical translation of NFκB-targeted therapies. NFκB pathway is a critical regulator of many cellular processes, and its inhibition can have unintended consequences. For example, NFκB pathway is involved in the regulation of inflammation, and its inhibition can increase the risk of infection. Moreover, it has opposing functions toward tumorigenesis. For instance, NFκB pathway is pivotal in orchestrating an appropriate immune response against cancer, yet concurrently, it can also promote the development of a tumor-promoting microenvironment by activating immunity-suppressive immune cells^19^. Therefore, targeting c-Rel provides a specific and coherent treating strategy. It coherently promotes tumor growth, both in tumor cells themselves or in immune cells, thus it won't create a dual effect that compromise the drug efficacies unlike targeting traditional NFκB members.

## 5. Conclusion

REL is a novel tumor oncogene in LUSC that contributes significantly to LUSC growth by facilitating cell proliferation and migration. Crucially, these cellular processes are orchestrated by the activation of the NFκB pathway. Our investigation elucidates the regulatory mechanisms of c-Rel in lung cancer and provides a promising foundation for the identification and development of novel therapeutic targets to combat LUSC.

## Supplementary Material

Supplementary figures and tables.
