# Supplementary material for: c-Rel is a Novel Oncogene in Lung Squamous Cell Carcinoma Regulating Cell Proliferation and Migration
Source: J Cancer. 2024 Mar 4;15(8):2329–39. doi: 10.7150/jca.93766 (PMC10937284; doi:10.7150/jca.93766)
Supplement: Supplementary file 1 — Supplementary figures and tables. [file jcav15p2329s1.pdf]

Supplementary Materials for

c-Rel is a novel oncogene in lung squamous cell carcinoma regulating cell proliferation and migration

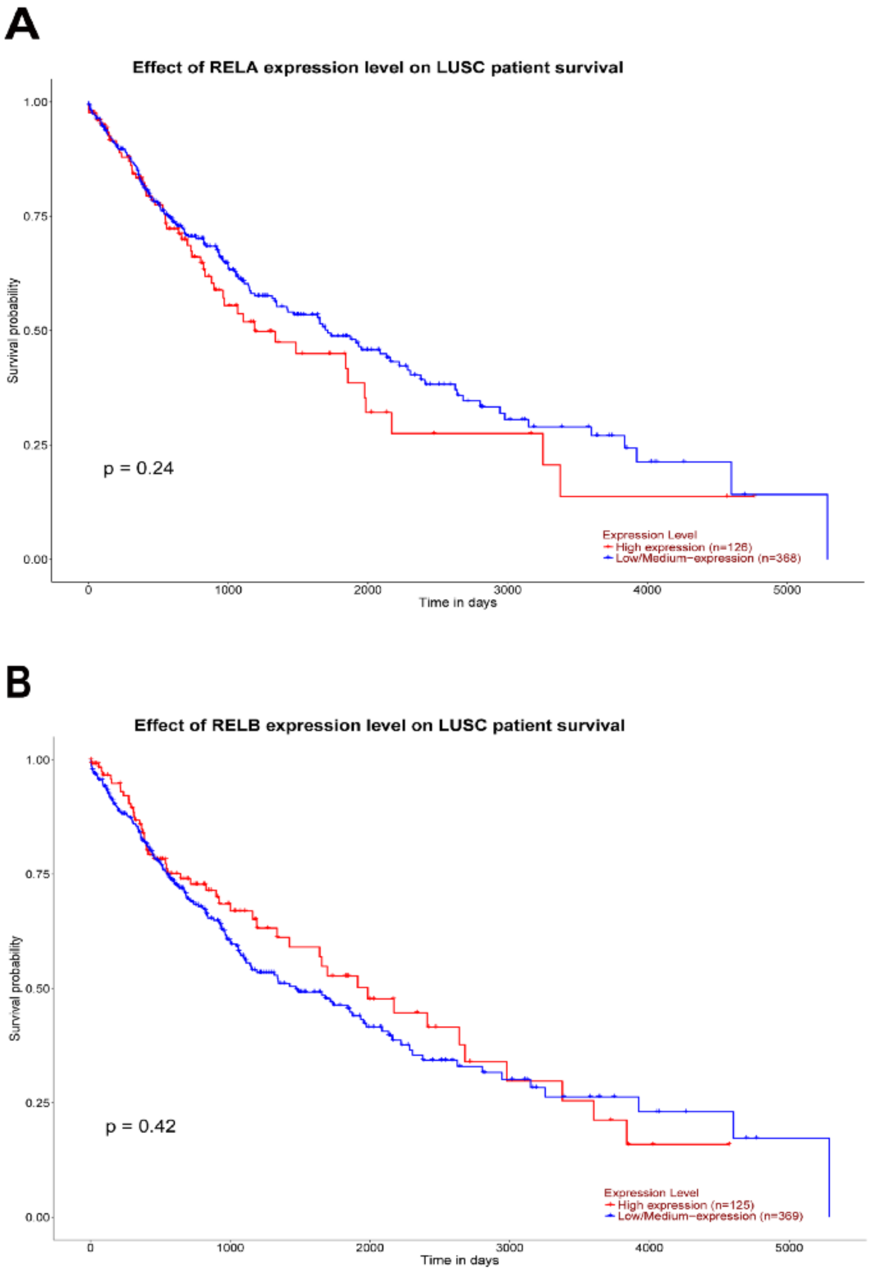

Supplementary figure S1. c-Rel was identified in a screening for oncogenes amplified in LUSC. (**A**) Comparative analysis of overall survival data derived from the TCGA database demonstrated no difference in survival rates between patients with low and high RelA expression levels. (**B**) Comparative analysis of overall

survival data derived from the TCGA database demonstrated no difference in survival rates between patients with low and high RelB expression levels.

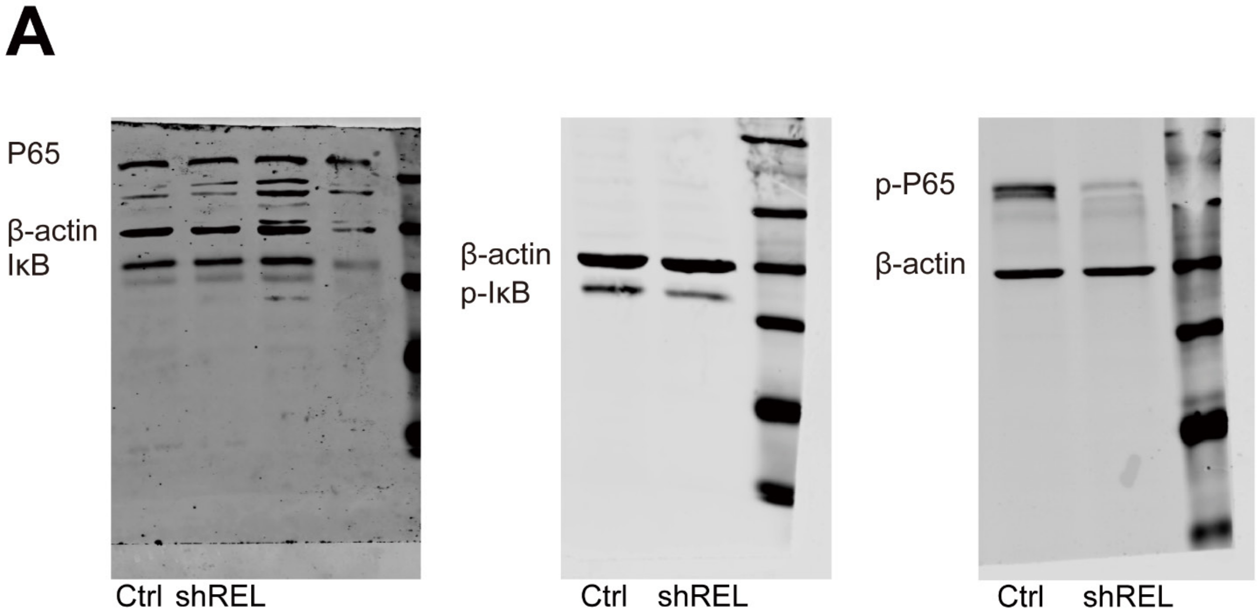

Supplementary figure S2. c-Rel regulates LUSC growth through activating NFκB pathway. (**A**) Original images of western blot for examining IκB, p- IκB, P65, and p-P65 protein level in NCI-H1703 cell upon c-Rel knockdown.

A

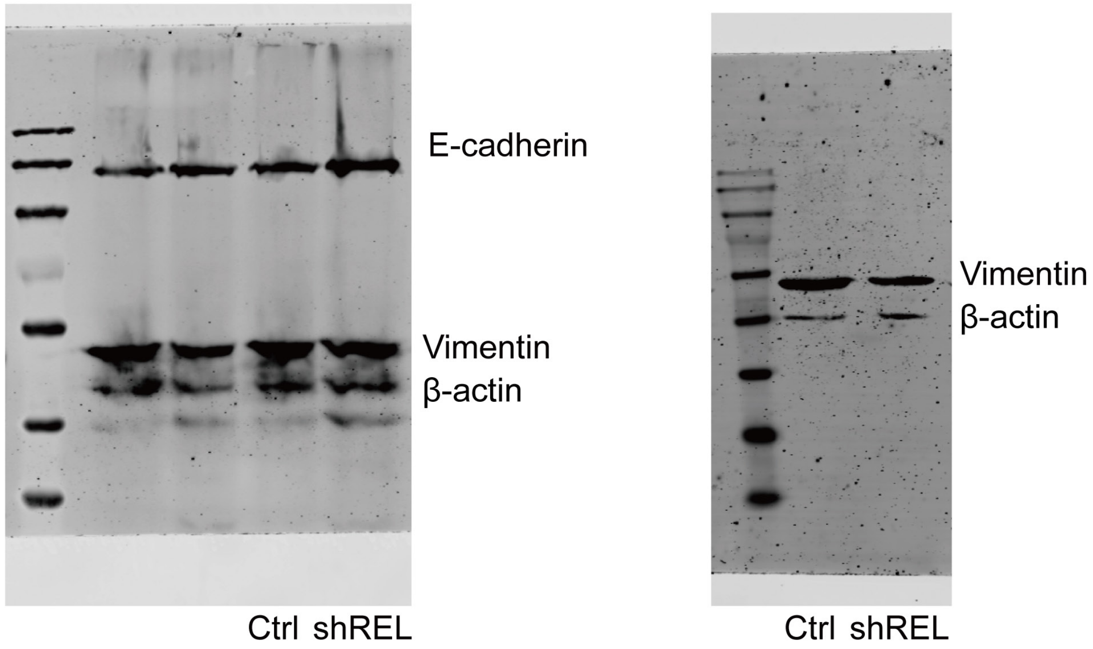

Supplementary figure S3. c-Rel promotes LUSC growth in vivo. (**A**) Original images of western blot for examining E-cadherin and vimentin protein level in the lungs of mice after injection of control and shREL LLC cells.

Supplementary table S1. Primers for Quantitative Real-time PCR.

Gene	Forward primer(5'-3')	Reverse primer(3'-5')
REL	AGTTGCGGAGACCTTCTGACCA	CGTGATCCTGGCACAGTTTCTG
RELA	TGAACCGAAACTCTGGCAGCTG	CATCAGCTTGCGAAAAGGAGCC
NFKB1A	TCCACTCCATCCTGAAGGCTAC	CAAGGACACCAAAAGCTCCACG
IL6	AGACAGCCACTCACCTCTTCAG	TTCTGCCAGTGCCTCTTTGCTG
TNFA	CTCTTCTGCCTGCTGCACTTTG	ATGGGCTACAGGCTTGTCACTC
ACTIN	CACCATTGGCAATGAGCGGTTC	AGGTCTTTGCGGATGTCCACGT

Supplementary table S2. 25 Tier 1 oncogenes were selected based on the Cancer Gene Census in COSMIC database.

SOX2	ETV5	FGFR1	PIK3CB	POLQ
PIK3CA	LPP	TERT	KAT6A	REL
TBL1XR1	TP63	CCND1	IKBKB	BCL11A
MECOM	BCL6	IL7R	EGFR	XPO1
MAP3K13	WWTR1	FOXL2	GATA2	CCNE1
